# Supplementary material for: Quadruple bonding between iron and boron in the BFe(CO)3− complex
Source: Nat Commun. 2019 Oct 17;10:4713. doi: 10.1038/s41467-019-12767-5 (PMC6797760; doi:10.1038/s41467-019-12767-5)
Supplement: Supplementary file 1 — Supplementary Information [file 41467_2019_12767_MOESM1_ESM.pdf]

Supplementary Information

Quadruple Bonding between Iron and Boron in the $BFe(CO)_3^-$ Complex

Chaoxian Chi, Jia-Qi Wang, Han-Shi Hu*, Yang-Yang Zhang, Wan-Lu Li,
Luyan Meng, Mingbiao Luo, Mingfei Zhou* & Jun Li*

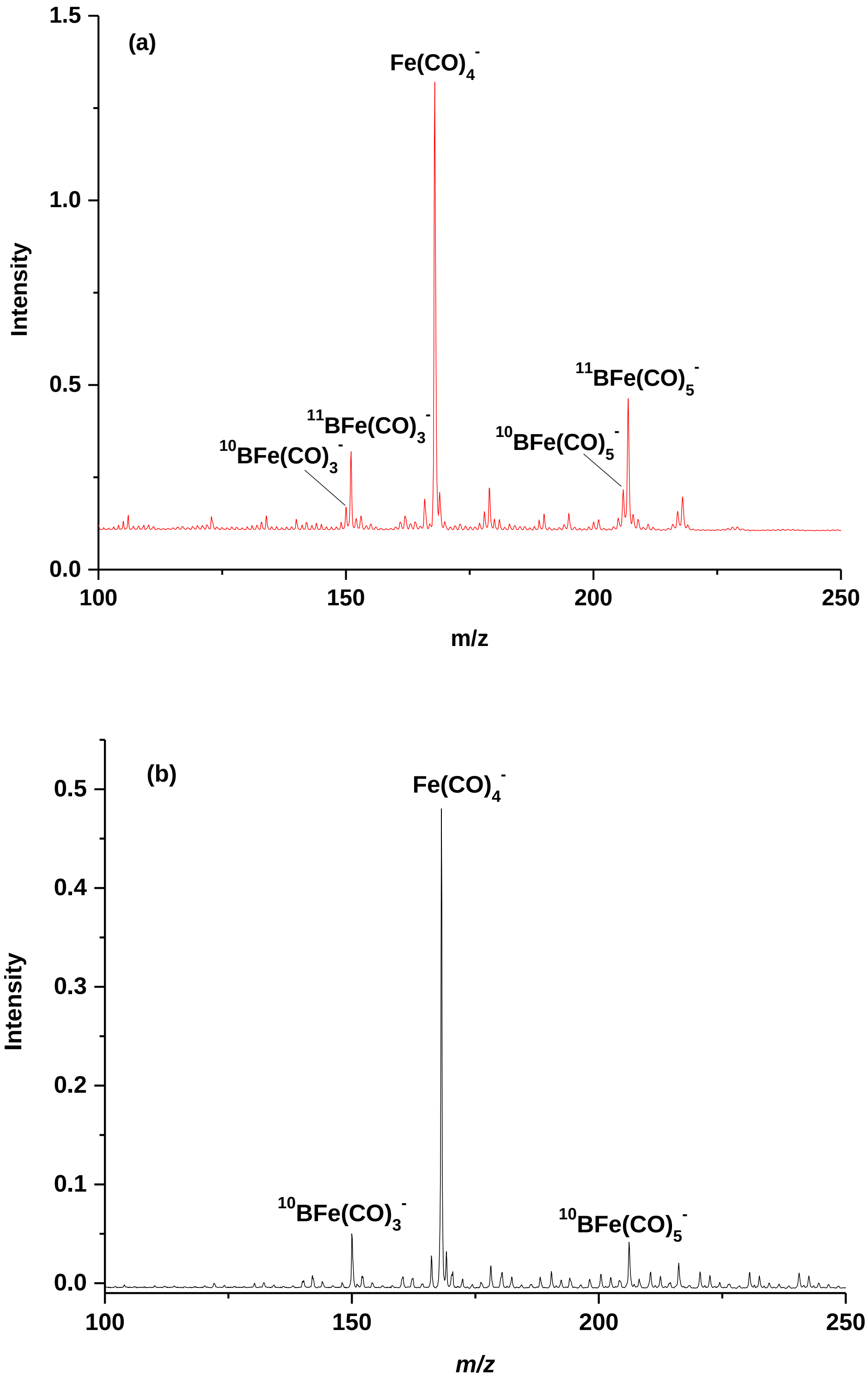

Supplementary Fig. 1 Mass spectra. The carbonyl anion complexes are formed by pulsed laser vaporization of (a) natural abundance boron target, and (b) Boron-10 enriched target in an expansion of helium seeded by 7% carbon monoxide with trace of iron carbonyl impurity.

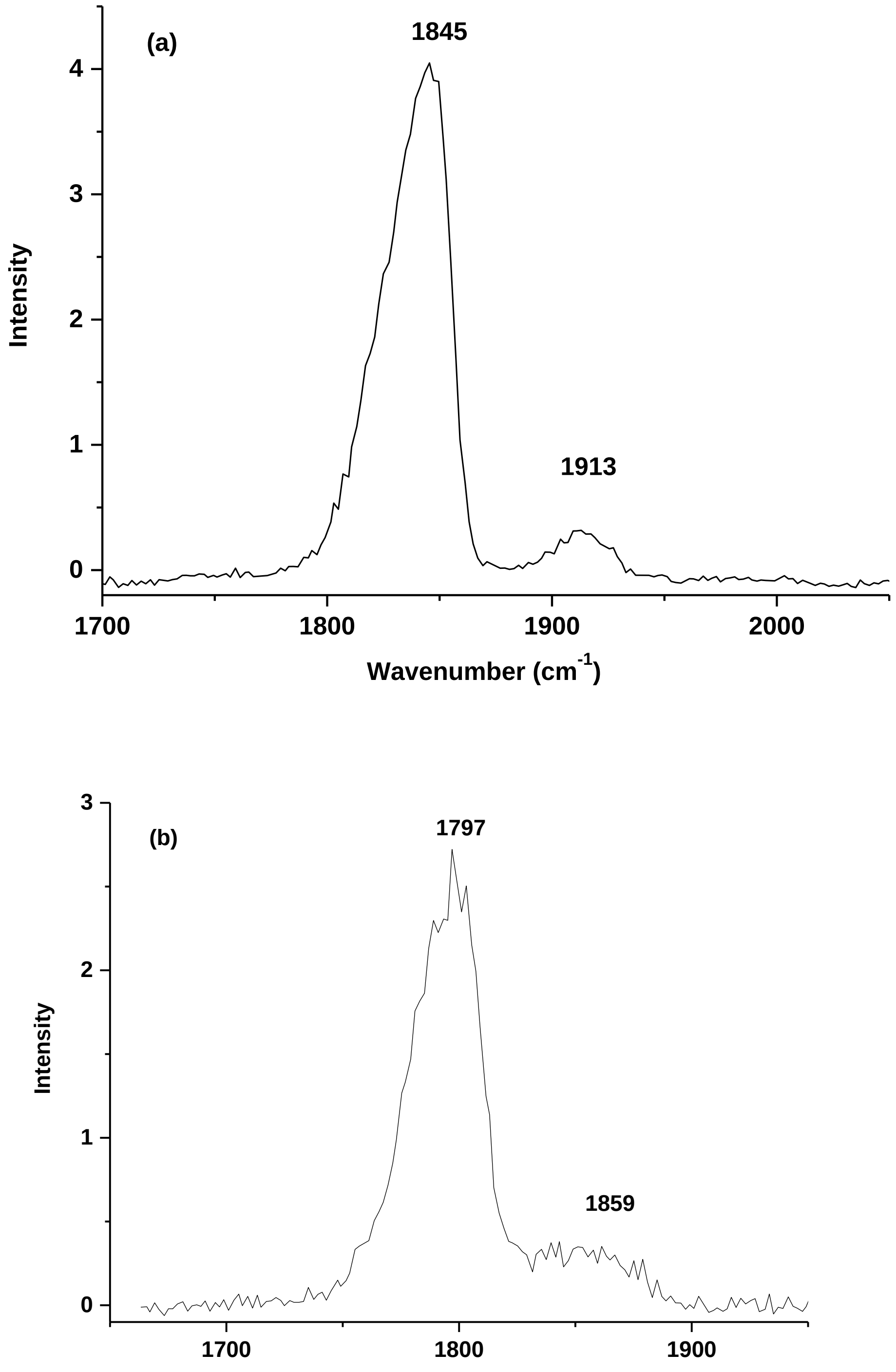

Supplementary Fig. 2 Infrared photodissociation spectra. (a) $^{10}BFe(CO)_3^-$ and (b) $^{11}BFe(^{13}CO)_3^-$.

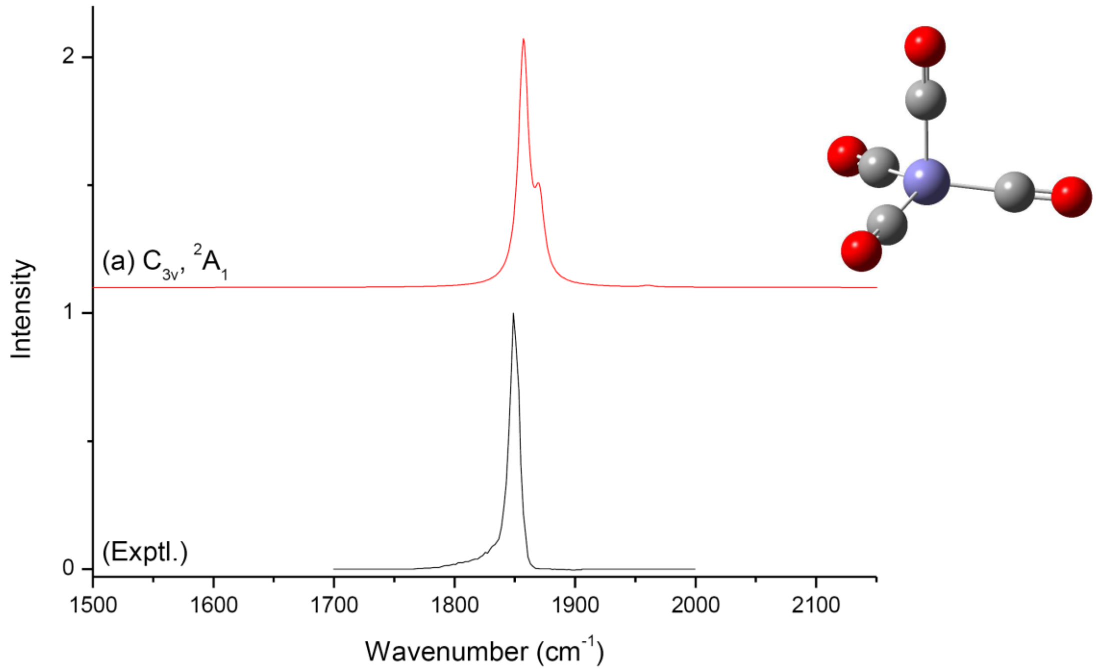

Supplementary Fig. 3 The experimental and simulated IR spectra of the $Fe(CO)_4^-$ anion. The predicted IR spectrum was obtained from scaled harmonic vibrational frequencies and intensities for the C_{3v} $^2A_1$ ground state of $Fe(CO)_4^-$ by applying Lorentzian line shape function with a 5 cm^{-1} full width at half-maximum.

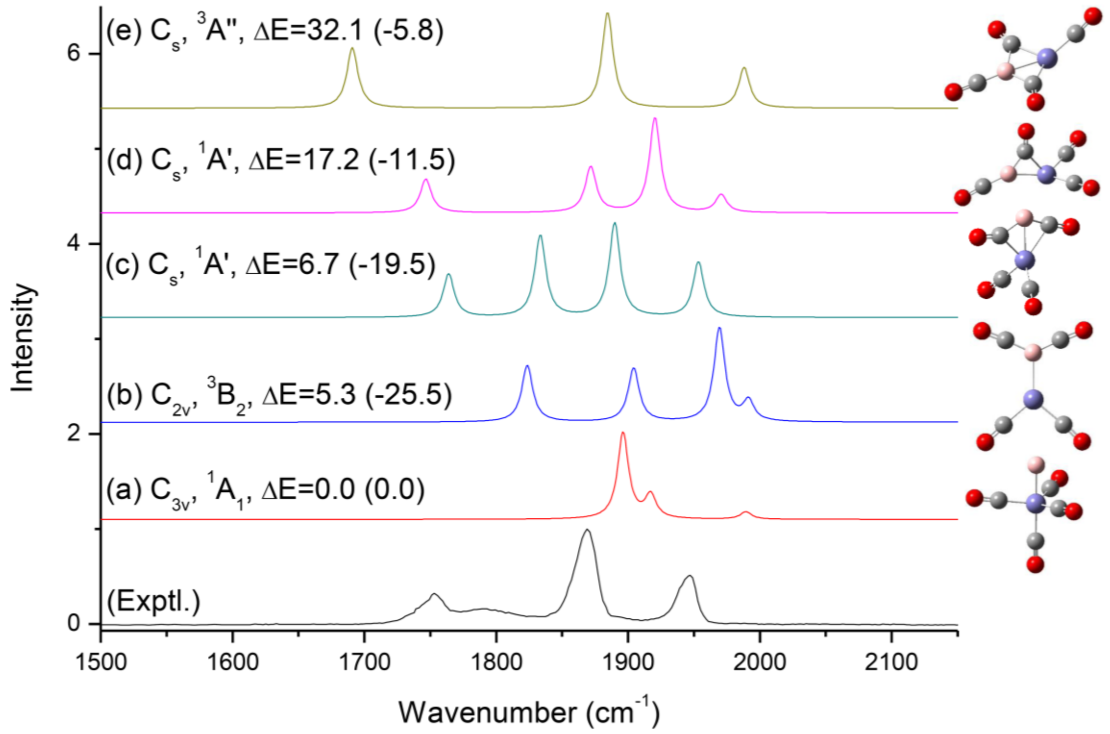

Supplementary Fig. 4 The experimental and simulated (B3LYP) IR spectra of the $BFe(CO)_4^-$ anion. The predicted IR spectra (a)-(e) were obtained from scaled harmonic vibrational frequencies and intensities for the five isomers of $BFe(CO)_4^-$ by applying Lorentzian line shape function with a 5 cm^{-1} full width at half-maximum. The relative energies at the DLPNO-CCSD(T) and B3LYP (in parentheses) levels are given in kcal mol^{-1}. The DLPNO-CCSD(T) calculations indicate that structure (a) is the most stable isomer, and structure (b) and (c) lie about 5.3 and 6.7 kcal mol^{-1} above structure (a). None of the simulated spectra of them agree with the experimental spectrum, suggesting that the observed $BFe(CO)_4^-$ anion is a mixture likely involving the lowest-lying isomers (a)-(c). The computed electronic energies at the B3LYP level clearly carry large error due to the inaccurate description of electron correlation, as discussed in *J. Chem. Theory. Comput.* 2016, 12, 1525-1533, DOI: 10.1021/acs.jctc.5b01040.

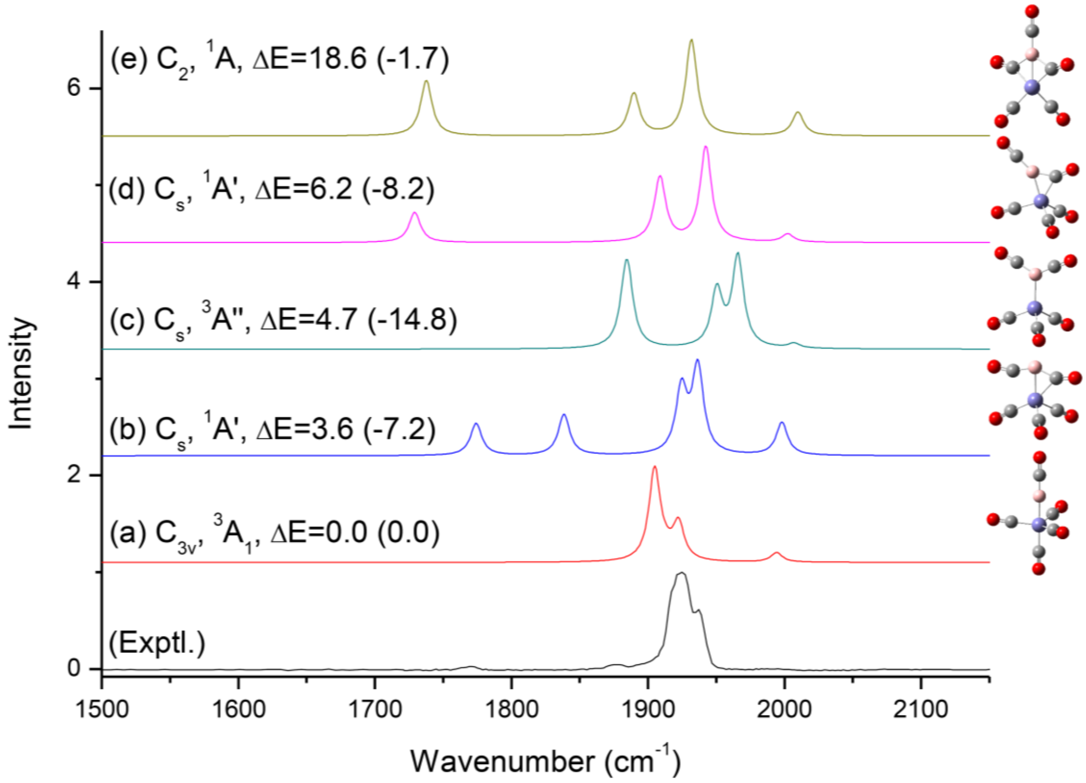

Supplementary Fig. 5 The experimental and simulated (B3LYP) IR spectra of the $BFe(CO)_5^-$ anion. The predicted IR spectra (a)-(e) were obtained from scaled harmonic vibrational frequencies and intensities for the five isomers of $BFe(CO)_5^-$ by applying Lorentzian line shape function with a 5 cm^{-1} full width at half-maximum. The relative energies at the DLPNO-CCSD(T) and B3LYP (in parentheses) levels are given in kcal mol^{-1}. The DLPNO-CCSD(T) calculations indicate that structure (a) is the most stable isomer, and structure (b), (c) and (d) lie only about 3.6, 4.7 and 6.1 kcal mol^{-1} above structure (a). The results suggest that the experimentally observed $BFe(CO)_5^-$ anion is mainly due to the most stable C_{3v} OCB-$Fe(CO)_4^-$ structure (a), likely with minor contribution from structures (b) and (c).

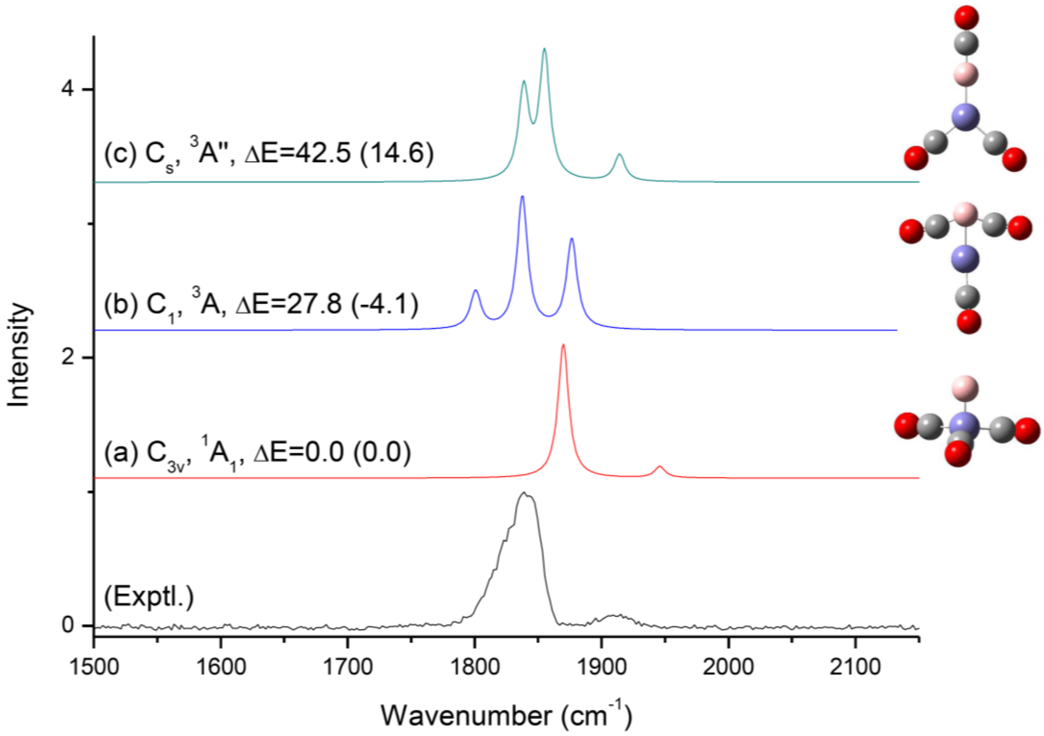

Supplementary Fig. 6 Experimental and simulated vibrational spectra of the $BFe(CO)_3^-$ anion in the carbonyl stretching frequency region. The simulated spectra were obtained from scaled harmonic frequencies and intensities for the three lowest-lying isomers of $BFe(CO)_3^-$ (C_{3v}, $^1A_1$), $(OC)_2BFe(CO)^-$ (C_1, ^3A) and $(OC)BFe(CO)_2^-$ (C_s, $^3A''$), respectively, by applying Lorentzian line shape function with a 5 cm^{-1} full width at half-maximum. The relative energies at the DLPNO-CCSD(T) and B3LYP (in parentheses) levels are given in kcal mol^{-1}.

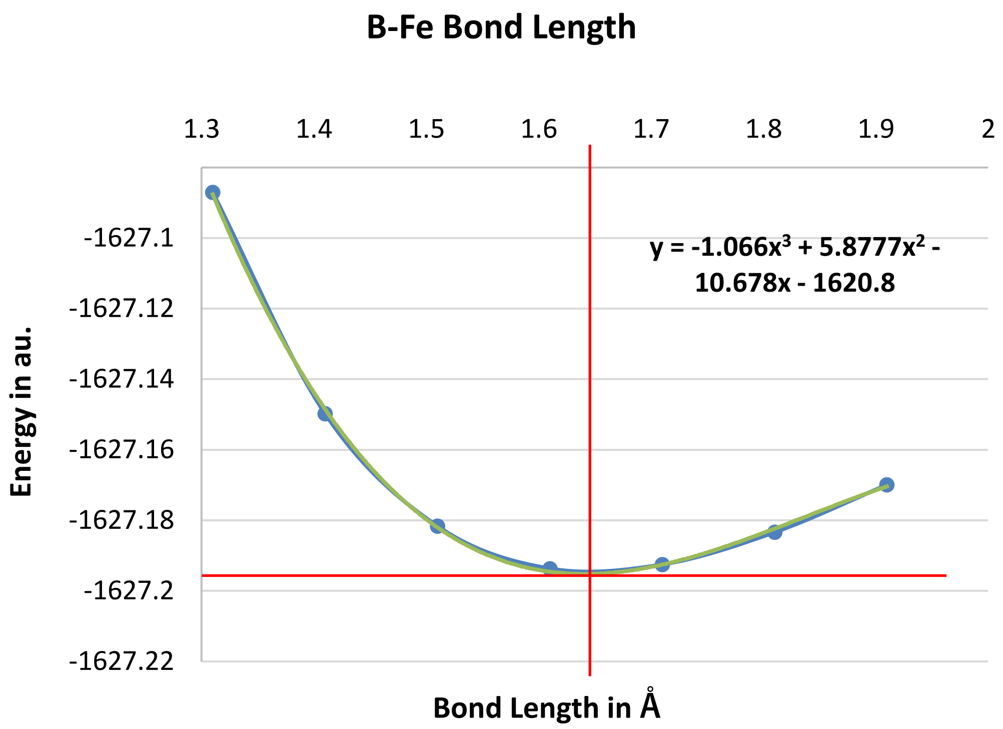

Supplementary Fig. 7 Potential energy curve for the ground state of $BFe(CO)_3^-$ at the DLPNO-CCSD(T) level of theory.

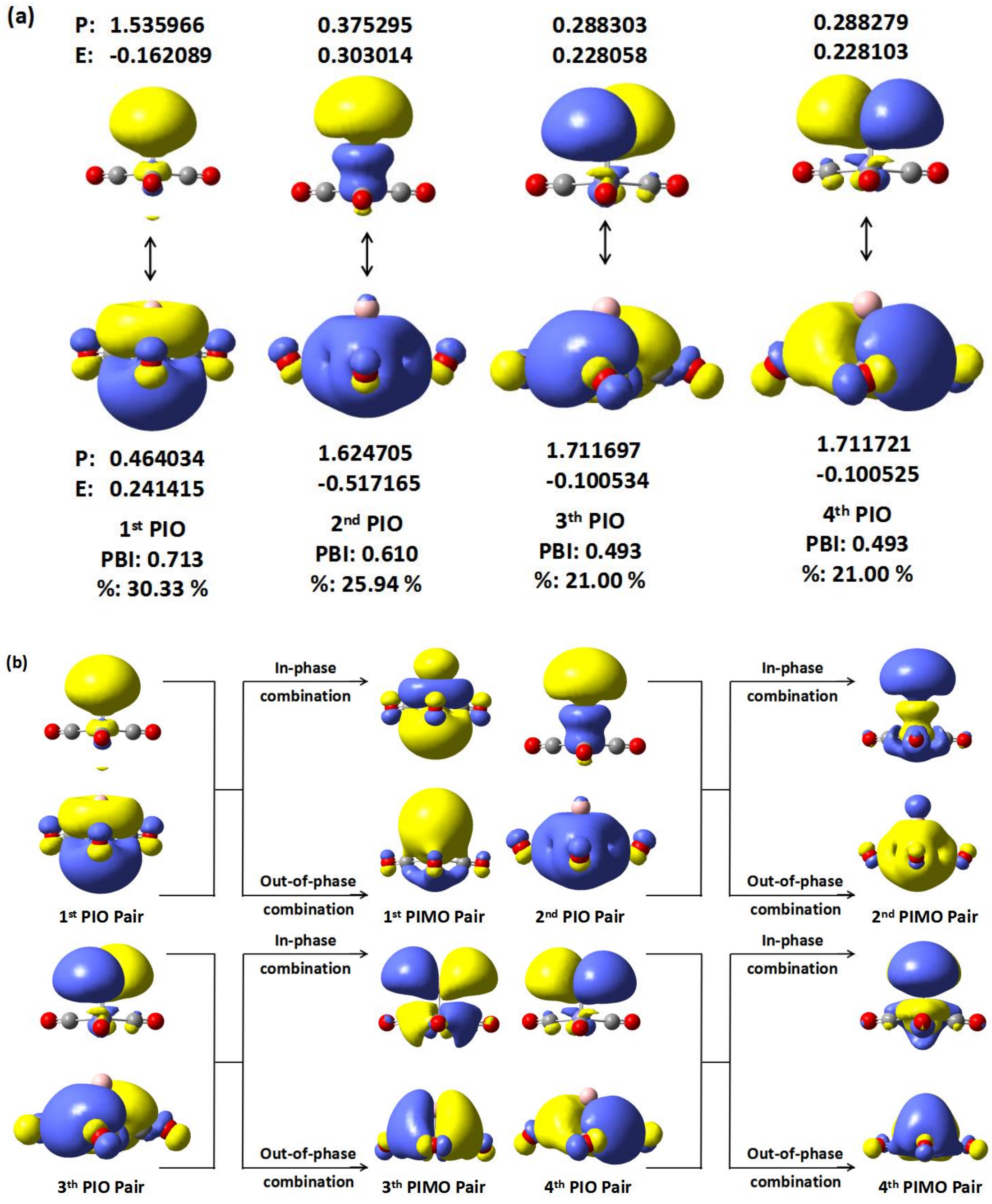

Supplementary Fig. 8 Results of PIO analysis on $BFe(CO)_3^-$ with B and $Fe(CO)_3$ as two fragments based on the M06-2X/aug-cc-pVTZ calculations. (a) The top four PIOs of each fragment. The orbital energies and populations (occupation numbers) are given as E and P, respectively, near each PIO. Given below each PIO pair are the PIO-based bond indices (abbreviated as PBI) and its contribution (as %) to the total interactions between two fragments (the contributions of all PIOs sum up to 100%). (b) The top four PIMO pairs and its relationship with the PIO pair. Note that the phase of each PIO is naturally paired up with its counterpart. Isovalue: 0.02 for all orbitals.

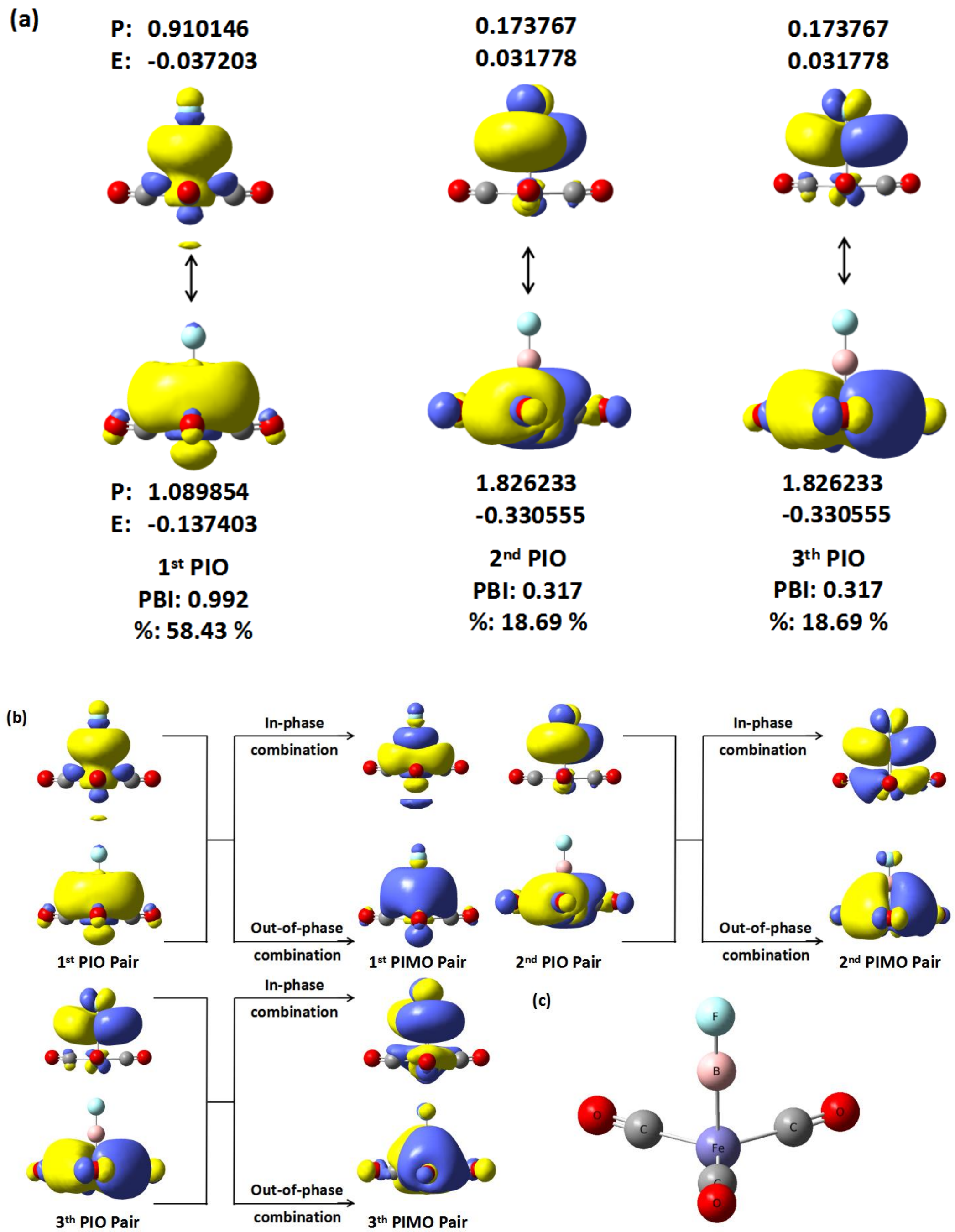

Supplementary Fig. 9 Results of PIO analysis on $FBFe(CO)_3$ with FB and $Fe(CO)_3$ as two fragments based on the M06-2X/aug-cc-pVTZ level. (a) The top three PIOs of each fragment. The orbital energies and populations (occupation numbers) are given as E and P, respectively, near each PIO. Given below each PIO pair are the PIO-based bond indices (abbreviated as PBI) and its contribution (as %) to the total interactions between two fragments (the contributions of all PIOs sum up to 100%). (b) The top three PIMO pairs and its relationship with the PIO pair. Note that the phase of each PIO is naturally paired up with its counterpart. Isovalue: 0.02

for all orbitals. (c) The scheme of fragmentation of $FBFe(CO)_3$ into FB and $Fe(CO)_3$ (fragments) carried out in the PIO analysis.

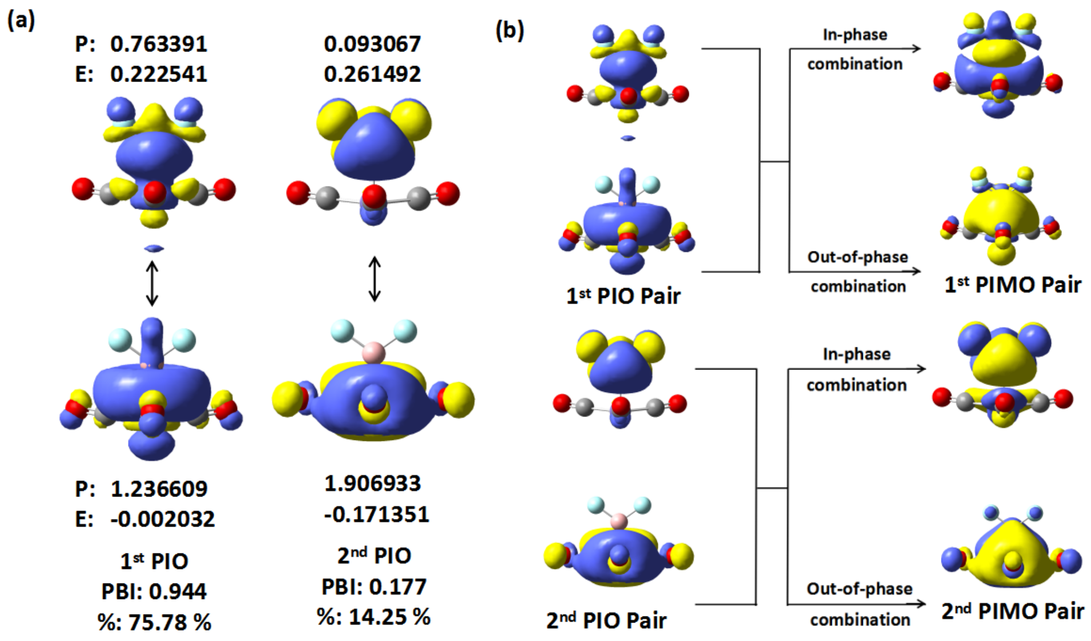

Supplementary Fig. 10 Results of PIO analysis on $F_2BFe(CO)_3^-$ with F_2B and $Fe(CO)_3$ as two fragments based on the M06-2X/aug-cc-pVTZ level. (a) The top two PIOs of each fragment. The orbital energies and populations (occupation numbers) are given as E and P, respectively, near each PIO. Given right each PIO pair are the PIO-based bond indices (abbreviated as PBI) and its contribution (as %) to the total interactions between two fragments (the contributions of all PIOs sum up to 100%). (b) The top two PIMO pairs and its relationship with the PIO pair. Note that the phase of each PIO is naturally paired up with its counterpart. Isovalue: 0.02 for all orbitals. (c) The scheme of fragmentation of $F_2BFe(CO)_3^-$ into F_2B and $Fe(CO)_3$ (fragments) carried out in the PIO analysis.

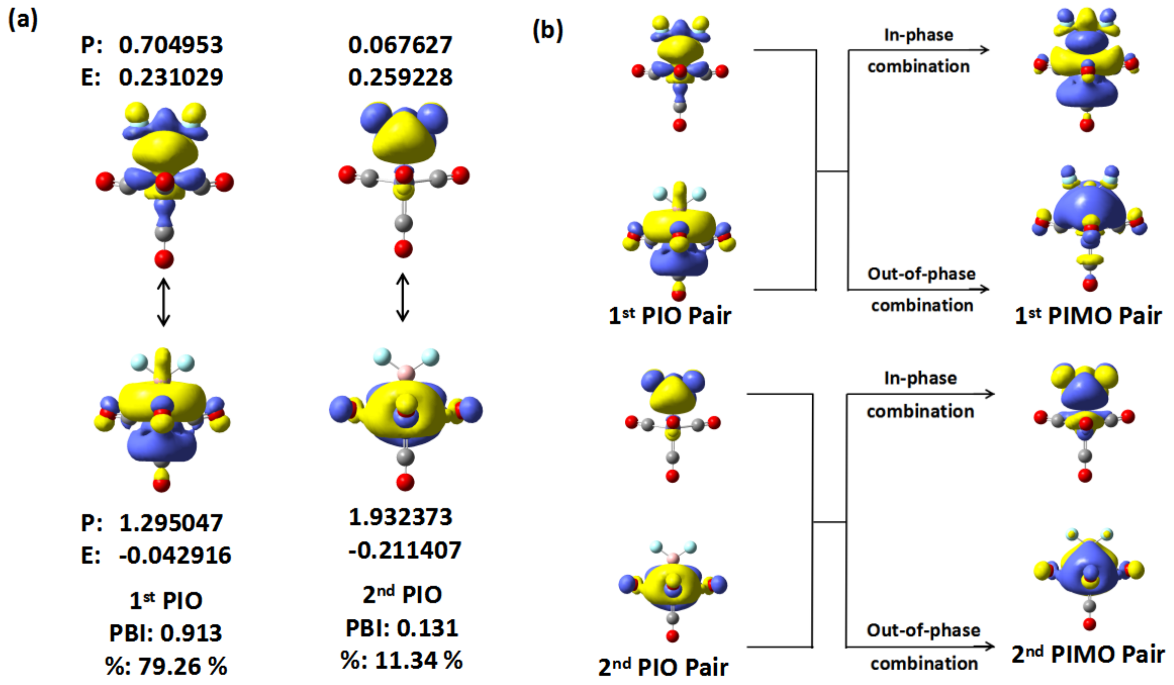

Supplementary Fig. 11 Results of PIO analysis on $F_2BFe(CO)_4^-$ with F_2B and $Fe(CO)_4^-$ as two fragments based on the M06-2X/aug-cc-pVTZ level. (a) The top two PIOs of each fragment. The orbital energies and populations (occupation numbers) are given as E and P, respectively, near each PIO. Given right each PIO pair are the PIO-based bond indices (abbreviated as PBI) and its contribution (as %) to the total interactions between two fragments (the contributions of all PIOs sum up to 100%). (b) The top two PIMO pairs and its relationship with the PIO pair. Note that the phase of each PIO is naturally paired up with its counterpart. Isovalue: 0.02 for all orbitals. (c) The scheme of fragmentation of $F_2BFe(CO)_4^-$ into F_2B and $Fe(CO)_4^-$ (fragments) carried out in the PIO analysis. (Note for the second PIO, the contribution is mainly from $Fe(CO)_4^-$ and very few in F_2B fragment (1.93 v.s. 0.07), indicating it is rather an Fe-B single bond.)

Type	1σ(Fe-B)	δ(Fe-C)	δ(Fe-C)	π(Fe-B)	π(Fe-B)	2σ(Fe-B)
**NOON**	**1.96**	**1.92**	**1.92**	**1.91**	**1.91**	**1.88**

NO

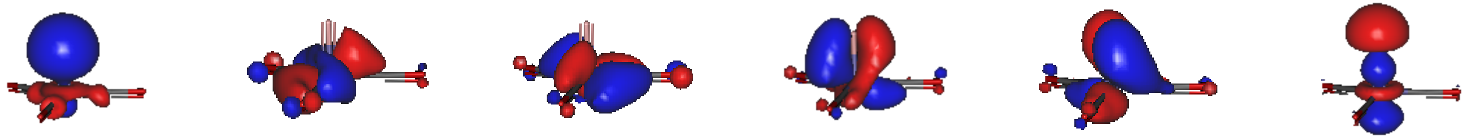

Type	1σ*(Fe-B)	δ*(Fe-C)	δ*(Fe-C)	π*(Fe-B)	π*(Fe-B)	2σ*(Fe-B)
**NOON**	**0.05**	**0.08**	**0.08**	**0.09**	**0.09**	**0.11**

NO

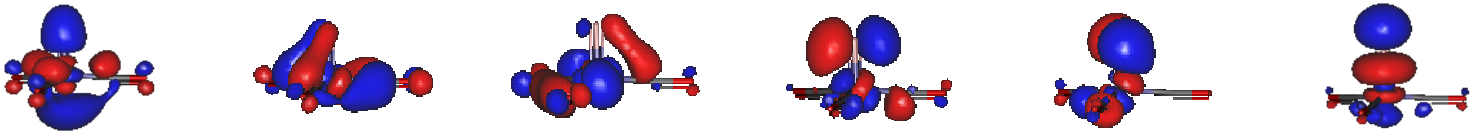

Supplementary Fig. 12 Natural orbitals (NOs) of the $BFe(CO)_3^-$ from CASSCF (12_e,12_o): natural orbital occupation numbers (NOON) and contour surfaces (0.05 au).

Supplementary Table 1 Calculated relative energies (kcal mol^{-1}) for different isomers of $BFe(CO)_3^-$ at the DFT/B3LYP/aug-cc-pVTZ and DLPNO-CCSD(T) (in parentheses) levels of theory.

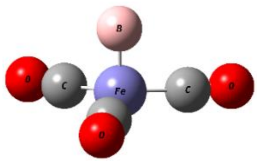	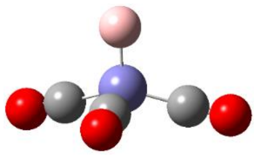	
**(a) C_{3v}, $^1A_1$, ΔE=0.0 (0.0)**	**(b) C_1, ^3A, ΔE=22.6**	
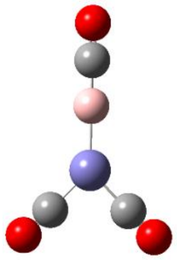	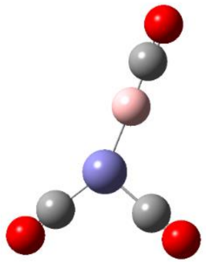	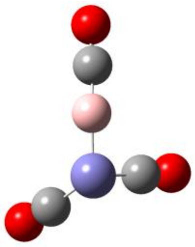
**(c) C_s, $^3A''$, ΔE=14.6 (42.5)**	**(d) C_s, $^5A''$, ΔE=20.2**	**(e) C_1, ^1A, ΔE=30.4**
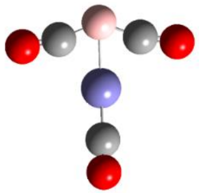	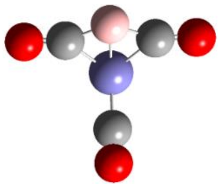	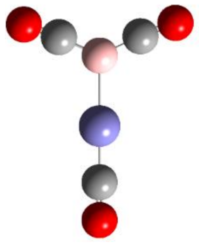
**(f) C_1, ^3A, ΔE=-4.1 (27.8)**	**(g) C_1, ^1A, ΔE=16.1**	**(h) C_1, ^3A, ΔE=21.1**

Supplementary Table 2 The B-Fe bond length (R in Å), AO contributions, relaxed force constant (Rfc) between B and Fe (N m^{-1}) and AdNDP analysis of the $BFe(CO)_3^-$, $FBFe(CO)_3$, $F_2BFe(CO)_3^-$, and $F_2BFe(CO)_4^-$ complexes at the M06-2X/TZ2P level.

<table>
<tr><th colspan="5">$BFe(CO)_3^-$</th></tr>
<tr><td>bond length</td><td colspan="4">1.61 Å</td></tr>
<tr><td>Rfc</td><td colspan="4">481 N m^{-1}</td></tr>
<tr><td rowspan="4">AO contribution</td><td>Orbital</td><td colspan="2">Isosurface (0.05)</td><td>AO contribution</td></tr>
<tr><td>σ</td><td colspan="2">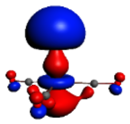</td><td>B: 26% $2p_z$ + 23% 2s
Fe: 28% $3d_{z^2}$ + 9% $4p_z$</td></tr>
<tr><td>π</td><td>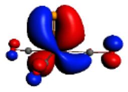</td><td>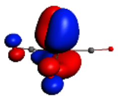</td><td>B: 10% $2p_{x/y}$
Fe: 68% $3d_{xz/yz}$</td></tr>
<tr><td>σ</td><td colspan="2">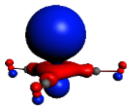</td><td>B: 40% 2s + 2% $2p_z$
Fe: 45% $3d_{z^2}$ + 3% $4p_z$</td></tr>
<tr><td rowspan="4">AdNDP</td><td>5c-2e
#\|e\|=1.99</td><td colspan="3">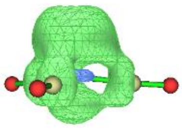</td></tr>
<tr><td>5c-2e
#\|e\|=1.96</td><td colspan="3">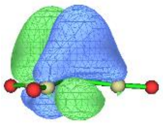</td></tr>
<tr><td>5c-2e
#\|e\|=1.96</td><td colspan="3">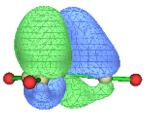</td></tr>
<tr><td>5c-2e
#\|e\|=1.96</td><td colspan="3">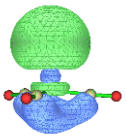</td></tr>
</table>

<table>
<tr><th colspan="4">$FBFe(CO)_3$</th></tr>
<tr><td>bond length</td><td colspan="3">1.81 Å</td></tr>
<tr><td>Rfc</td><td colspan="3">220 N m^{-1}</td></tr>
<tr><td>AO</td><td>orbital</td><td>Isosurface (0.05)</td><td>AO contribution</td></tr>
</table>

contribution	π_p	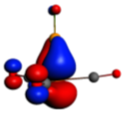	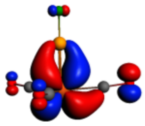	B: 9% $2p_{x/y}$<br>Fe: 82% $3d_{xz/yz}$
	σ	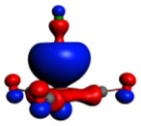		B: 38% 2s + 13% $2p_z$<br>Fe: 27% $3d_{z^2}$ + 2% 4s + 3% $4p_z$
AdNDP	2c-2e<br>#|e|=1.97	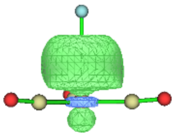		
	5c-2e<br>#|e|=1.98	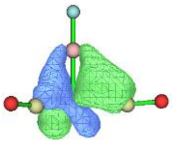		
	5c-2e<br>#|e|=1.98	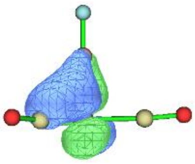		

**$F_2BFe(CO)_3^-$**			
bond length	1.94 Å		
Rfc	158 N m^{-1}		
AO contribution	orbital	Isosurface (0.05)	AO contribution
	π	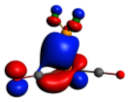	B: 7% 2s + 6% $2p_z$<br>Fe: 54% $3d_{xz}$ + 10% $3d_{z^2}$
	σ	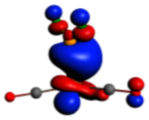	B: 14% 2s + 12% $2p_z$<br>Fe: 27% $3d_{xz}$ + 25% $3d_{z^2}$ + 4% $4p_z$
AdNDP	5c-2e<br>#|e|=1.99	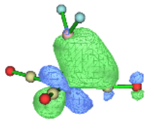	
	2c-2e<br>#|e|=1.92	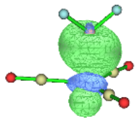	

<table>
<tr><th colspan="4">$F_2BFe(CO)_4^-$</th></tr>
<tr><td>bond length</td><td colspan="3">2.02 Å</td></tr>
<tr><td>Rfc</td><td colspan="3">140 N m^{-1}</td></tr>
<tr><td rowspan="2">AO
contribution</td><td>orbital</td><td>Isosurface (0.05)</td><td>AO contribution</td></tr>
<tr><td>σ</td><td>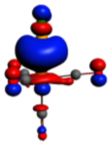</td><td>B: 20% 2s + 20% $2p_z$
Fe: 21% $3d_{z^2}$ + 9% $4p_z$</td></tr>
<tr><td>AdNDP</td><td>6c-2e
#|e|=1.92</td><td colspan="2">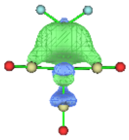</td></tr>
</table>

Supplementary Table 3 AO contributions (in %) in KS-MOs of $BFe(CO)_3^-$ at the M06-2X/TZ2P level of theory.

$^1A_1$ C_{3v}-**$BFe(CO)_3^-$**							
	B			Fe			CO
	2p	2s	3p	4s	3d	4p	2s2p
HOMO 11e	10				44	14	32
HOMO-1 $14a_1$	26	23			28	9	37
HOMO-2 10e	10				68		17
HOMO-3 $13a_1$		40	2	3	45	3	7

Supplementary Table 4 Charge analysis in $BFe(CO)_3^-$ with M06-2X/TZ2P method.

	Mulliken charge	Hirshfeld	VDD
B	-0.05	-0.20	-0.27
Fe	-0.55	-0.25	-0.24
C	0.33	0.04	0.05
O	-0.46	-0.22	-0.22
C	0.33	0.04	0.05
O	-0.46	-0.22	-0.22
C	0.33	0.04	0.05
O	-0.46	-0.22	-0.22

Supplementary Table 5 Energy decomposition analysis of $BFe(CO)_3^-$ at the M06-2X/TZ2P level (Isosurfaces = 0.005 au). Energy values are given in kcal mol^{-1}.

fragments	B: $s^2p_z^1$ $Fe(CO)_3^-$: $^2A_1$		
ΔE_{int}	-127.5		
ΔE_{Pauli}	392.8		
ΔE_{elstat}	-296.9 (57.1%)		
ΔE_{orb}	-223.4 (42.9%)		
	α	β	α + β
$\Delta E_{orb(\sigma p)}$	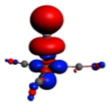 -59.6	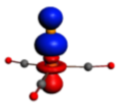 -36.1	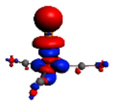 -95.7 (42.8%)
$\Delta E_{orb(\pi\perp)}$	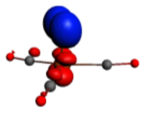 -27.0	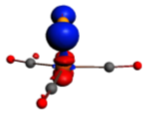 -25.1	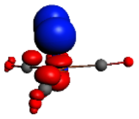 -52.1 (23.3%)
$\Delta E_{orb(\pi//)}$	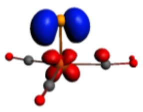 -27.0	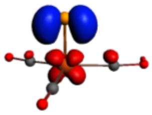 -25.1	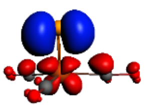 -52.1 (23.3%)
$\Delta E_{orb(\sigma s)}$	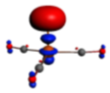 -8.8	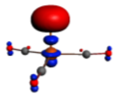 -9.6	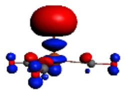 -18.4 (8.2%)
E_{prep}	2.9		
$E_{bonding}$	-124.6		

[a]The value in parentheses gives the percentage contribution to the total attractive interactions $\Delta E_{elstat}+\Delta E_{orb}$. [b] The value in parentheses gives the percentage contribution to the total orbital interactions ΔE_{orb}. [c] $\Delta E_{tot\text{-}bonding} = \Delta E_{prep} + \Delta E_{int}$

Supplementary Table 6 The scale factors of PBE, B3LYP, and M06-2X.

	free	PBE	B3LYP	M06-2X
CO frequency (cm^{-1})	2143	2123	2205	2282
ratio		1.01	0.97	0.94

*The scale factors are taken from the ratio of the experimental frequency (2143 cm^{-1}) and the calculated harmonic frequency (based on the corresponding DFT method) for free CO.
