# Supplementary material for: Quadruple bonding between iron and boron in the BFe(CO)3− complex
Source: Nat Commun. 2019 Oct 17;10:4713. doi: 10.1038/s41467-019-12767-5 (PMC6797760; doi:10.1038/s41467-019-12767-5)
Supplement: Supplementary file 2 — Supplementary Data [file 41467_2019_12767_MOESM2_ESM.pdf]

Supplementary Data

Quadruple Bonding between Iron and Boron in the $BFe(CO)_3^-$ Complex

Chaoxian Chi, Jia-Qi Wang, Han-Shi Hu*, Yang-Yang Zhang, Wan-Lu Li,
Luyan Meng, Mingbiao Luo, Mingfei Zhou* & Jun Li*

Supplementary Data 1 Cartesian Coordinates (Å) of the species in Supplementary Figs. 3-6 calculated at the B3LYP/aug-cc-pVTZ level. Calculated energies are the sum of electronic and zero-point energies.

(a) C_{3v}-$Fe(CO)_4^-$-$^2A_1$, E = -1717.379872 Hartree

Fe	0.00000000	0.00000000	0.22920200
C	0.00000000	1.77468500	0.51470500
O	0.00000000	2.92770800	0.66149500
C	1.53692200	-0.88734300	0.51470500
O	2.53546900	-1.46385400	0.66149500
C	-1.53692200	-0.88734300	0.51470500
O	-2.53546900	-1.46385400	0.66149500
C	0.00000000	0.00000000	-1.55826000
O	0.00000000	0.00000000	-2.71878300

(b) C_{3v}-$BFe(CO)_4^-$-$^1A_1$, E= -1742.149513 Hartree

Fe	0.00000000	0.00000000	0.14320600
C	0.00000000	1.76377800	0.37031700
C	1.52747600	-0.88188900	0.37031700
C	-1.52747600	-0.88188900	0.37031700
C	0.00000000	0.00000000	-1.70337800
B	0.00000000	0.00000000	2.04046500
O	0.00000000	2.91170800	0.51979100
O	-2.52161300	-1.45585400	0.51979100
O	2.52161300	-1.45585400	0.51979100
O	0.00000000	0.00000000	-2.85576100

(c) C_{2v}-$BFe(CO)_4^-$-$^3B_2$, E= -1742.190076 Hartree

Fe	0.00000000	0.00000000	0.57947400
B	0.00000000	0.00000000	-1.59262700
C	0.00000000	1.26836100	-2.21317600
C	0.00000000	-1.38150900	1.75932200
C	0.00000000	1.38150900	1.75932200
O	0.00000000	2.34732500	-2.66287200
O	0.00000000	-2.22906300	2.55931200
O	0.00000000	2.22906300	2.55931200
C	0.00000000	-1.26836100	-2.21317600
O	0.00000000	-2.34732500	-2.66287200

(d) C_s-$BFe(CO)_4^-$-$^1A'$, E= -1742.180610 Hartree

Fe	-0.23871300	0.02590400	0.00000000
B	0.74161200	1.78812300	0.00000000
C	1.57267400	0.55029000	0.00000000
C	-0.65193700	2.01990500	0.00000000
C	-0.35121500	-1.16108600	1.30207300
O	2.64609800	0.02425700	0.00000000
O	-1.79509100	2.38092400	0.00000000
O	-0.35121500	-1.89648100	2.19647500
C	-0.35121500	-1.16108600	-1.30207300
O	-0.35121500	-1.89648100	-2.19647500

(e) C_s-$BFe(CO)_4^-$-$^1A'$, E= -1742.167871 Hartree

Fe	0.46987800	-0.23872800	0.00000000
B	-0.32477700	1.50733700	0.00000000
C	-1.29524600	0.35038700	0.00000000

C	0.57877700	-1.44451200	1.26821300
O	-2.41962700	-0.05377900	0.00000000
O	0.57877700	-2.21272700	2.13887900
C	0.57877700	-1.44451200	-1.26821300
C	-0.11599800	2.89686500	0.00000000
O	0.57877700	-2.21272700	-2.13887900
O	0.12822200	4.04434100	0.00000000

(f) C_s-$BFe(CO)_4^-$-$^3A''$, E= -1742.158825 Hartree

C	-0.51674800	2.50067500	0.00000000
C	-0.12962200	0.30692700	1.42438300
C	-0.12962200	0.30692700	-1.42438300
O	-0.12962200	0.60048100	-2.58426500
O	-0.66520400	3.66155300	0.00000000
O	-0.12962200	0.60048100	2.58426500
C	0.85728300	-2.33812500	0.00000000
O	1.63465100	-3.20411600	0.00000000
B	-0.28925100	1.10777000	0.00000000
Fe	-0.18165800	-0.90248000	0.00000000

(g) C_{3v}-$BFe(CO)_5^-$-$^3A_1$, E= -1855.564594 Hartree

Fe	0.00000000	0.00000000	0.44035900
B	0.00000000	0.00000000	-1.50688600
C	0.00000000	1.76984400	0.22722300
C	1.53273000	-0.88492200	0.22722300
C	-1.53273000	-0.88492200	0.22722300
O	0.00000000	2.91625200	0.07509600
O	2.52554800	-1.45812600	0.07509600
O	-2.52554800	-1.45812600	0.07509600

C	0.00000000	0.00000000	2.23646900
C	0.00000000	0.00000000	-2.91284300
O	0.00000000	0.00000000	3.38982000
O	0.00000000	0.00000000	-4.10844100

(h) C_s-$BFe(CO)_5^-$-$^1A'$, E= -1855.575997 Hartree

Fe	0.07491700	-0.06304000	0.00000000
C	0.10891900	-1.25342000	1.31088900
C	0.10891900	-1.25342000	-1.31088900
C	-0.21796200	2.15677700	0.00000000
C	-1.93304300	0.01267100	0.00000000
C	1.78171700	0.52878500	0.00000000
O	-2.84623900	-0.76473900	0.00000000
O	2.88536300	0.85636000	0.00000000
O	0.10891900	-2.00175100	2.18824900
O	0.10891900	-2.00175100	-2.18824900
O	0.49011600	3.11709000	0.00000000
B	-1.40315400	1.36980200	0.00000000

(i) C_s-$BFe(CO)_5^-$-$^3A''$, E= -1855.588222 Hartree

Fe	-0.31580300	-0.38081300	0.00000000
B	1.14405400	1.23774100	0.00000000
C	2.51494700	0.89183200	0.00000000
C	-2.07344500	0.15026200	0.00000000
C	-0.31644900	-1.54723300	1.42118800
O	3.62612700	0.52996100	0.00000000
O	-3.17846300	0.48840500	0.00000000
O	-0.31644900	-2.27903700	2.31549000
C	-0.31644900	-1.54723300	-1.42118800

C	0.64142500	2.55939000	0.00000000
O	-0.31644900	-2.27903700	-2.31549000
O	0.15903900	3.62349900	0.00000000

(j) C_s-$BFe(CO)_5^-$-$^1A'$, E= -1855.577738 Hartree

Fe	-0.20266600	-0.35019900	0.00000000
B	0.73471700	1.34542900	0.00000000
C	1.64635300	0.14640100	0.00000000
C	-1.81130400	0.48513900	0.00000000
C	-0.24238600	-1.55505900	1.29475500
O	2.77038300	-0.27443700	0.00000000
O	-2.81688000	1.05121600	0.00000000
O	-0.24238600	-2.30859200	2.17100200
C	-0.24238600	-1.55505900	-1.29475500
C	0.70228300	2.75846800	0.00000000
O	-0.24238600	-2.30859200	-2.17100200
O	0.69131400	3.92774100	0.00000000

(k) C_2-$BFe(CO)_5^-$-^1A, E= -1855.567242 Hartree

Fe	0.00000000	0.00000000	0.56437300
C	0.00000000	1.43185400	-0.58598800
C	0.00000000	-1.43185400	-0.58598800
C	1.13315300	0.60354300	1.76895900
C	-1.13315300	-0.60354300	1.76895900
O	0.00787400	2.58629500	-0.87236800
O	-0.00787400	-2.58629500	-0.87236800
O	1.92493400	0.92016700	2.55364400
O	-1.92493400	-0.92016700	2.55364400
B	0.00000000	0.00000000	-1.40215100

C	0.00000000	0.00000000	-2.81600100
O	0.00000000	0.00000000	-3.98287600

(l) C_{3v}-BFe(CO)$_3^-$-$^1A_1$, E= -1628.774462 Hartree

C	0.00000000	1.76832500	-0.17922900
C	1.53141400	-0.88416200	-0.17922900
C	-1.53141400	-0.88416200	-0.17922900
O	2.52871200	-1.45995300	-0.32844400
O	0.00000000	2.91990600	-0.32844400
O	-2.52871200	-1.45995300	-0.32844400
Fe	0.00000000	0.00000000	0.08925800
B	0.00000000	0.00000000	1.75761500

(m) C_1-BFe(CO)$_3^-$-^3A, E= -1628.781075 Hartree

B	1.81791800	0.00095500	-0.26987400
Fe	-0.23274600	-0.00023600	0.36656300
C	1.30627900	1.31671400	-0.19661600
O	1.11891900	2.50137900	-0.14758300
C	1.30798300	-1.31541400	-0.19666400
O	1.12182500	-2.50026800	-0.14767500
C	-1.98194600	-0.00091000	-0.04558100
O	-3.09475600	-0.00123300	-0.39825500

(n) C_s-BFe(CO)$_3^-$-$^3A''$, E= -1628.751187 Hartree

Fe	0.00000000	0.30370900	0.00000000
B	0.00189600	-1.66594200	0.00000000
C	1.37133700	1.47991400	0.00000000
O	2.25970100	2.23089700	0.00000000

C	-1.37485900	1.47557900	0.00000000
O	-2.26560100	2.22378000	0.00000000
C	0.00334500	-3.10387100	0.00000000
O	0.00484800	-4.28923300	0.00000000

Supplementary Data 2 Cartesian Coordinates (Å) of the species listed in Table 1.

PBE/TZ2P, C_{3v}-$BFe(CO)_3^-$-$^1A_1$, E=-2.27649 Hartree

C	-0.879481	1.523306	-0.037796
C	-0.879481	-1.523306	-0.037796
C	1.758963	0.000000	-0.037796
O	-1.463464	-2.534794	0.098523
O	-1.463464	2.534794	0.098523
O	2.926928	0.000000	0.098523
Fe	0.000000	0.000000	-0.284892
B	0.000000	0.000000	-1.973733

B3LYP/TZ2P, C_{3v}-$BFe(CO)_3^-$-$^1A_1$, E=-2.70364 Hartree

C	-0.880848	1.525673	-0.034835
C	-0.880848	-1.525673	-0.034835
C	1.761695	0.000000	-0.034835
O	-1.457085	-2.523745	0.114144
O	-1.457085	2.523745	0.114144
O	2.914170	0.000000	0.114144
Fe	0.000000	0.000000	-0.301609
B	0.000000	0.000000	-1.966567

M06-2X/TZ2P, C_{3v}-$BFe(CO)_3^-$-$^1A_1$, E=-3.45905 Hartree

C	-0.876873	1.518788	-0.039301
C	-0.876873	-1.518788	-0.039301
C	1.753745	0.000000	-0.039301
O	-1.452422	-2.515668	0.030068
O	-1.452422	2.515668	0.030068

O	2.904844	0.000000	0.030068
Fe	0.000000	0.000000	-0.248266
B	0.000000	0.000000	-1.856532

Supplementary Data 3 Cartesian Coordinates (Å) of the species listed in Supplementary Table 1 at the B3LYP/aug-cc-pVTZ level. Calculated energies are the sum of electronic and zero-point energies.

(a) C_{3v}-$BFe(CO)_3^-$-$^1A_1$, E= -1628.774462 Hartree

C	0.00000000	1.76832500	-0.17922900
C	1.53141400	-0.88416200	-0.17922900
C	-1.53141400	-0.88416200	-0.17922900
O	2.52871200	-1.45995300	-0.32844400
O	0.00000000	2.91990600	-0.32844400
O	-2.52871200	-1.45995300	-0.32844400
Fe	0.00000000	0.00000000	0.08925800
B	0.00000000	0.00000000	1.75761500

(b) C_1-$BFe(CO)_3^-$-^3A, E= -1628.738418 Hartree

C	1.67839600	0.71098200	-0.26530200
C	0.00141600	-1.49216200	-0.17135300
C	-1.67966400	0.70913400	-0.26536200
O	0.00222500	-2.62806100	-0.40907600
O	2.77550100	0.99061700	-0.52421500
O	-2.77705100	0.98788300	-0.52409400
Fe	-0.00032600	0.23626600	0.23302000
B	0.00043700	-0.10283000	1.96253300

(c) C_s-$BFe(CO)_3^-$-$^3A''$, E= -1628.751187 Hartree

Fe	0.00000000	0.30370900	0.00000000
B	0.00189600	-1.66594200	0.00000000
C	1.37133700	1.47991400	0.00000000
O	2.25970100	2.23089700	0.00000000

C	-1.37485900	1.47557900	0.00000000
O	-2.26560100	2.22378000	0.00000000
C	0.00334500	-3.10387100	0.00000000
O	0.00484800	-4.28923300	0.00000000

(d) C_s-$BFe(CO)_3^-$-$^5A''$, E= -1628.742288 Hartree

Fe	0.00000000	0.42036100	0.00000000
B	1.03483500	-1.38999400	0.00000000
C	0.01230700	2.24563900	0.00000000
O	-0.04462800	3.40877500	0.00000000
C	-1.79825900	0.12394000	0.00000000
O	-2.94670500	-0.06700900	0.00000000
C	1.77698200	-2.61160100	0.00000000
O	2.35128800	-3.65767600	0.00000000

(e) C_1-$BFe(CO)_3^-$-^1A, E= -1628.725994 Hartree

Fe	0.59257100	-0.01999100	0.74257100
B	-1.14054100	-0.35833600	0.45881800
C	0.37007600	1.45621600	-0.10547600
O	0.22291800	2.40384200	-0.77065600
C	1.74680600	-0.89906200	-0.25019500
O	2.36307100	-1.44823300	-1.07388500
C	-2.44264000	-0.49404700	-0.15536500
O	-3.55468700	-0.71400900	-0.47229800

(f) C_1-$BFe(CO)_3^-$-^3A, E= -1628.781075 Hartree

B	1.81791800	0.00095500	-0.26987400
Fe	-0.23274600	-0.00023600	0.36656300

C	1.30627900	1.31671400	-0.19661600
O	1.11891900	2.50137900	-0.14758300
C	1.30798300	-1.31541400	-0.19666400
O	1.12182500	-2.50026800	-0.14767500
C	-1.98194600	-0.00091000	-0.04558100
O	-3.09475600	-0.00123300	-0.39825500

(g) C_1-BFe$(CO)_3^-$-^{1}A, E= -1628.748743 Hartree

B	0.00016200	-1.38007100	-0.78273200
Fe	0.00001800	0.04902300	0.60980100
C	1.31349000	-0.93305800	-0.31051500
O	2.51820000	-0.92488300	-0.21582400
C	-1.31326600	-0.93329800	-0.31043700
O	-2.51798200	-0.92553700	-0.21579900
C	-0.00010600	1.69464700	0.01264800
O	-0.00046700	2.68242000	-0.60479400

(h) C_1-BFe$(CO)_3^-$-^{3}A, E= -1628.740852 Hartree

B	-1.36573900	-0.00023400	-0.00027700
Fe	0.84832500	0.00001800	-0.00001000
C	-1.95858700	-1.27734200	-0.00014900
O	-2.36921100	-2.37386600	0.00015000
C	-1.95355900	1.27918500	0.00004500
O	-2.35980500	2.37737300	0.00007200
C	2.61294500	-0.00206000	0.00008700
O	3.79994600	-0.00325800	-0.00000200
